# Externalities in appropriation: responses to probabilistic losses

**DOI:** 10.1007/s10683-017-9511-x

**Published:** 2017-02-10

**Authors:** Esther Blanco, Tobias Haller, James M. Walker

**Affiliations:** 1 Department of Public Finance, University of Innsbruck, Universitaetsstrasse 15, 6020 Innsbruck, Austria; 2 Department of Economics, Indiana University, Wylie Hall 105, Bloomington, IN 47405 USA; 3 The Ostrom Workshop, Indiana University, Bloomington, IN USA

**Keywords:** Social dilemma, Laboratory experiment, Endogenous externality, Strategic uncertainty, Ecosystem services, D70, D81, H41, C90

## Abstract

Using an appropriation game setting, we examine individual responses to changes in a groups’ vulnerability to a probabilistic loss (*L*) of a public good. The probabilistic loss parameter entails losing 10, 50 or 90% of the value of the public good that is maintained through cooperation, where the likelihood of the loss decreases in total group cooperation. By design, the expected marginal net benefits to an individual and the expected harm to others depends endogenously on the individuals’ expectations of group cooperation and exogenously on the magnitude of the loss parameter. We find that individual cooperation is greater when forecasts of total group cooperation are greater and where the magnitude of the probabilistic loss is larger. There is, however, an interesting asymmetry in responses by two subgroups. Subjects who are pessimistic regarding total group cooperation decrease cooperation the higher the magnitude of the probabilistic loss and their decisions are tied systematically to changes in their expectations of other’s cooperation. On the other hand, subjects who are optimistic regarding total group cooperation are found to be more cooperative, but their decisions are not systematically tied to changes in expectations of others’ cooperation.

**Electronic supplementary material:**

The online version of this article (doi:10.1007/s10683-017-9511-x) contains supplementary material, which is available to authorized users.

## Introduction

A growing number of experimental studies focus on issues related to probabilistic losses associated with the provision or maintenance of public goods. Motivated by issues of climate change, several previous studies have examined the behavioral response to variations in *exogenous* probabilities of group losses (Milinski et al. 2008, 2011; Barrett and Dannenberg 2012). Other contributions, more relevant to this study, examine *endogenous* probabilistic losses. In particular, Dickinson (1998) and Gangadharan and Nemes (2009) examine provision-point public goods settings where the probability of provision of a public good increases in contributions. In addition, Walker and Gardner (1992) and Blanco et al. (2016b) explore the relevance of endogenous probabilistic losses in appropriation game settings.

An important example of the relevance of endogenous probabilistic losses in appropriation settings relates to the provision or maintenance of ecosystem services, which have the characteristics of public goods in that they yield positive externalities to a population. In this context, probabilistic loss externalities are relevant in a wide collection of settings (see TEEB 2010) where increasing pressure by resource users results in an increased likelihood that a major ecosystem disturbance occurs and compromises the capacity of the ecosystem to generate ecosystem services or even to survive. The vulnerability of ecosystems to appropriation pressures is dependent upon a number of factors, including the geographical location, the ecosystem network, and the level of biodiversity. Similar issues of vulnerability and probabilistic losses apply to the provision of public goods, for example mitigation investments to dampen the effects of climate change.

In the context of an appropriation setting, this experimental study examines how subjects respond to changes in the *magnitude* of an endogenous probabilistic loss of a public good, where the probability of occurrence of the loss decreases with greater cooperation. Specifically, we examine loss parameters that entail losing 10, 50 or 90% of the value of the public good maintained through cooperation. The study makes several important contributions to the social dilemma literature. First, while previous experimental research has compared the response of subjects to endogenous probabilities of public good provision to settings with exogenous probabilities (Dickinson 1998; Gangadharan and Nemes 2009) and to settings without a probabilistic component (Gangadharan and Nemes 2009; Blanco et al. 2016b), the experiments reported here are the first to address the response of subjects to manipulations in the magnitude of the endogenous probabilistic component. Thus, we provide the first results on the quantitative response to the *magnitude* of endogenous losses rather than just to the *existence* of the endogenous component.

Secondly, to our knowledge, no previous social dilemma study has identified the asymmetry in behavior we observe between subjects who are optimistic about group performance and those who are pessimistic. On average, we show that own cooperation increases with forecasts of total group cooperation (and the corresponding marginal incentives to cooperate), and the treatment effects show that the quantitative response is greater the larger the magnitude of the loss parameter. Moreover, our results provide a novel insight by discovering significant differences in the response to variations in the loss parameter depending on whether subjects are pessimistic or optimistic about group behavior. Pessimistic subjects reduce cooperation the higher the magnitude of the loss parameter and their decisions are tied systematically to changes in the marginal incentives that correspond to their expectations of others’ appropriation. Optimistic subjects are more cooperative, but their decisions are not systematically tied to changes in marginal incentives that correspond to changes in expectations of others’ appropriation.

These novel results add to the emergent experimental literature that explores individual differences in the responses to marginal incentives and reciprocity (see for example, Brandts and Schram 2001; Fischbacher et al. 2001; Goeree et al. 2002; Brandts et al. 2004; Blanco et al. 2016a, b). These studies, like ours, address decisions in a menu game setting where subjects report cooperation levels for variations in marginal incentives or others’ cooperation. This attribute of the design allows us to examine within subject decisions in regard to how they respond to changes in game parameters. Moreover, like most of these studies (Fischbacher et al. 2001; Goeree et al. 2002; Blanco et al. 2016a, b), we report one-shot decisions. This allows us to abstract from group dynamics related to strategic play across decision rounds, and thus avoid the complexity of modeling subject’s responses to the dynamics of a repeated game. This type of setting is reminiscent of individual decisions in large group settings where there is limited or no knowledge of decisions by others and where group dynamics play little role in decision making.

In the games examined, we use a “take some” frame^1^ where appropriation leads to (1) deterministic losses, by reducing the value of a shared group resource, and to (2) endogenous probabilistic losses, where greater appropriation increases the probability that the shared resource faces an additional loss in value.^2^

Given the existence of an endogenous probabilistic loss, the expected value of the shared resource to an individual and the expected harm to others from appropriation is endogenously defined by the first order beliefs of others’ appropriation and exogenously defined through parameter variations in the magnitude of the probabilistic loss. By eliciting subjects’ expectations of other’s behavior, the experimental design allows for estimation of the relationship between changes in expected marginal incentives and appropriation decisions. In addition, for control purposes, the experimental design includes a “benchmark game” without the possibility of a probabilistic loss and fixed marginal incentives to appropriate. Using subjects’ decision in this game as a measure for “baseline cooperation”, we are able to examine individual responses to the addition of a probabilistic loss and to the changes in the magnitude of that loss.^3^

The studies cited above that focus on endogenous probabilistic losses vary in regard to how they approach the issue that expected marginal incentives change as group behavior changes. More specifically, Walker and Gardner (1992) focus on game continuation, and not on individual subject responses to expectations of marginal incentives. Both Dickinson (1998) and Gangadharan and Nemes (2009) focus on expected per capita return of contributions, based on one-period lagged behavior, implicitly assuming that expectations of current round group contributions are based on behavior in the previous round. Blanco et al. (2016b) introduces the deterministic and probabilistic degradation games used in this study and investigate the response to variations in subjects’ private benefits in settings without probabilistic losses and in settings with a probabilistic loss of 50% of the shared resource. This previous paper explicitly links expected value of marginal net benefits to subjects’ forecast of other group members’ appropriation. In contrast to the present study, that study does not examine the response to changes in the magnitude of the probabilistic loss nor does it examine differences in individual responses for optimistic and pessimistic subjects on group performance, as defined herein.^4^

## Decision settings and parameters

The experimental design included four one-shot decisions from a menu of games (part A), an incentivized first-order belief-elicitation task related to each of the games (part B), a risk aversion task (part C) and a dictator donation to charities (part D). In part A, incentives in all games are measured in Experimental Currency Units (ECUs). In these games, groups of *n* = 4 individuals face allocation decisions between a “Group Fund” and an “Individual Fund.” Each four member group begins with a Group Fund endowment of *w* = 100 tokens, where every token left in the Group Fund has a value of *g* = 2 ECUs. Each individual begins the game with 0 tokens allocated to their Individual Fund. Individuals privately decide how many tokens to move from the Group Fund which are then placed in their Individual Fund, with a maximum appropriation limit of *e* = 25 tokens per individual. Each token an individual *i* moves from the Group Fund, in a given treatment condition *j*, yields a private benefit increasing the value of his/her Individual Fund by *h* = 1 ECU. Each token left in the Group Fund has a value of *g/n* = 0.5 ECUs for every member of the group and thus appropriation generates a *deterministic degradation* to the group of *g*. Concurrently, appropriation generates a *probabilistic degradation*, implemented as a hazard rate that depends on the aggregate number of tokens appropriated from the Group Fund. Subjects confront a fractional loss *L* of the total value remaining in the Group Fund after all decisions are final. The endogenous probability of this loss occurring is $\left( {p\sum\nolimits_{i = 1}^{n} {z_{i} } } \right)$, where *p* = 0.01 the fractional increase in the probability associated with each token appropriated from the Group Fund. The feasible range of values of $p \in \left[ {0,1} \right]$ and $L \in \left[ {0,1} \right]$.

Letting $z_{ij}$ denote the amount individual *i* appropriates from the Group Fund in treatment *j*, Eq. (1) presents the payoff to individual *i* in ECUs. The probabilistic degradation externality is described in the last component of Eq. (1).

1 $$ \pi_{i}^{j} = hz_{ij} + \frac{g}{n}w - \frac{g}{n}Z - \left( {\frac{g}{n}} \right)\left[ {L_{j} \cdot \left( {p{\text{Z}}} \right)\left( {w - {\text{Z}}} \right)} \right] $$

where $Z = \sum\nolimits_{i = 1}^{n} {z_{ij} }$, and $p{\text{Z}} \le 1$. Ceteris paribus, the experimental design varies *L* across games in Part A, with *L* = 0.10, 0.50 and 0.90, and *p* = 0.01 in all cases.^5^ We refer to these treatment conditions as *L10*, *L50* and *L90*, and the benchmark game, where *L* = 0 as *L0*. A total of 111 subjects participated in these sessions.^6^

The instructions for each game in Part A, as well as quizzes to check subjects’ understanding of the games, were presented sequentially (see the Electronic Supplementary Material). As in Brandts and Schram (2001), it was the subjects’ choice to determine the order in which he/she made decisions in the games of part A. Importantly, at any point during decision-making in part A, subjects had the opportunity to review and change any of the choices they had already made. After all participants had time to finalize their decisions, the experimenter announced the end of part A, after which no one was allowed to change their decisions.

Part B was an incentivized belief elicitation task following Croson (2007), in which subjects were asked to report a forecast of the average per-person appropriation level for the other members of their group for each of the four games in part A. Subjects learned of the details of part B only after completing part A, with no feedback of results from part A. While making their forecasts, subjects could refer to a copy of their own decision-making sheet from part A.

Part C was a risk elicitation task that was a modified version from Dohmen et al. (2010), with the stake sizes used in Balafoutas et al. (2012). In this task subjects had to choose between a certain payment or a lottery yielding 5 Euros or 0 Euros, each with a 50% probability. Subjects made a total of 10 decisions, where the amount they received in the certain payment increased from 0.5 to 5 Euros in 50 cent intervals.

Part D was a dictator task with charities as recipients, where subjects had to allocate 3 Euros between themselves and one (or several) of eight charities offered to them. The decision sheet included a list of the charities as well as a short description of their mission. In order to circumvent the issue that some subjects might prefer to donate to one of the charities following the experiment, subjects were informed that the experimenter would increase the amount a subject allocated to the charities by 25%.

After finishing part D, subjects answered a short questionnaire. Payments were based on one of the games in each of the parts A, B, and C, and the amount of money subjects kept for themselves in part D. All drawings used for determining the games for computing experiment earnings were made in public. Subjects were paid in private in cash.^7^

## Expected marginal incentives

**Fig. 1:**
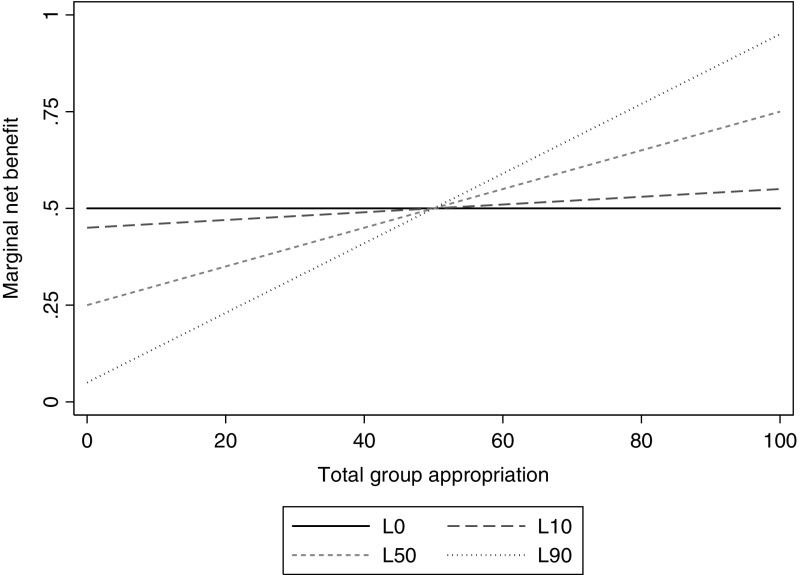
Marginal net benefits as a function of aggregate group appropriation

Based on the payoff functions given in Eq. (1), the marginal net benefit ($MNB_{i}^{j}$) of appropriation for individual *i* in treatment *j* is:

2 $$ MNB_{i}^{j} = \frac{{\partial \pi_{i}^{j} }}{{\partial z_{i} }} = h - \frac{g}{n} - \frac{g}{n}L_{j} p \cdot \left( {w - 2{\text{Z}}} \right) $$

where $p{\text{Z}} \le 1$. Notice, with *p* = 0.01 in all games, the probabilistic nature of the game implies that the magnitude of $MNB_{i}^{j}$ depends on aggregate group appropriation *Z*, and the parameter *L*. Thus, based on differences in first order beliefs of others’ behavior, subjects facing the same parameter values will face different marginal incentives to appropriate.^8^ Table 1 displays the specific functional relation between $MNB_{i}^{j}$ and aggregate group appropriation for each of the treatment conditions *j* = *L0, L10, L50* and *L90*. Figure 1 displays the value of $MNB_{i}^{j}$ at each possible level of group appropriation, as well as illustrating how it changes across treatment conditions.

**Table 1:** Decision settings: parameters and marginal net benefits

Decision setting	*L*	*p*	Marginal net benefit functions
*L0* (benchmark game)	0	0.01	$MNB_{i}^{L0} = 0.5$
*L10*	0.1	0.01	$MNB_{i}^{L10} = 0.45 + 0.001\cdot{\text{Z}}$
*L50*	0.5	0.01	$MNB_{i}^{L50} = 0.25 + 0.005\cdot{\text{Z}}$
*L90*	0.9	0.01	$MNB_{i}^{L90} = 0.05 + 0.009\cdot{\text{Z}}$

Parameters *n* = 4, *w* = 100, *e* = 25, *h* = 1 are constant in all games

Note that, for any value of *L* in the range [0, 1], the unique Nash equilibrium for self-interested payoff-maximizing agents is to appropriate at capacity. This follows from the observation that, given a maximum group capacity to appropriate of 100, the $MNB_{i}^{j}$ is positive for any value of *L* in the range [0, 1].

### **Hypothesis 1**

Self-interested payoff-maximizing agents appropriate at $z_{i} = 25$ tokens in all treatment conditions.

However, a broad range of previous research on social dilemma settings has shown that subjects make decisions that reflect complex and diverse motivations beyond simple self-income maximization (see research summarized in Camerer 2003; Camerer and Fehr 2006; Ostrom and Walker 2003). Some but not all of these motivations support models where subjects respond systematically to the private benefits of their actions (internal returns) and the magnitude of externalities imposed on others (external return) (see for example Goeree et al. 2002). The literature also provides support for models where decision makers follow other regarding preferences that are not sensitive to changes in magnitudes of externalities imposed on others, such as the concept of “warm glow” as introduced by Andreoni (1990), and examined in Palfrey and Prisbrey (1997) among others.

If subjects were to respond solely to marginal net benefits, how would we expect behavior to change with respect to the benchmark game in the treatment conditions? Referring back to Fig. 1, first note that as compared to the constant $MNB_{i}^{L0} = 0.5$ in the *L0* game, the expected marginal net benefit increases (decreases) for expectations of group appropriation above (below) a critical threshold of 50 tokens in all treatment conditions. Further, note that the range of values of $MNB_{i}^{j}$ as a function of group appropriation (vertical axis) increases with the size of *L*. In particular, $MNB_{i}^{L0} = 0.5$, $MNB_{i}^{L10} \in \left[ {0.45, 0.55} \right]$, $MNB_{i}^{L50} \in \left[ {0.25, 0.75} \right]$, $MNB_{i}^{L90} \in \left[ {0.05, 0.95} \right]$. Thus, across treatment conditions, the influence of first order beliefs of group appropriation on the expected magnitude of $MNB_{i}^{j}$ increases with increases in *L*.

### **Hypothesis 2**

Subjects responding to marginal net benefits increase (decrease) appropriation in the probabilistic settings as compared to the benchmark setting if expected total group appropriation is above (below) a threshold of 50 tokens.

### **Hypothesis 3**

Subject responses to changes in treatment conditions are more pronounced for larger loss parameters *L*.

Of course, as noted above, some subjects may also respond to incentives beyond their own pecuniary return. As discussed, appropriation by subject *i* creates a deterministic and probabilistic negative externality on other group members. More specifically, the marginal harm to each other group member −*i* from appropriation by subject *i*, $MH_{ - i}^{j}$, is based on the last two components in Eq. (2):

3 $$ MH_{ - i}^{j} = \frac{{\partial \pi_{ - i}^{j} }}{{\partial z_{i} }} = \frac{{\left( {n - 1} \right)g}}{n} + \frac{{\left( {n - 1} \right)g}}{n}L_{j} p \cdot \left( {w - 2{\text{Z}}} \right) $$

As shown, $MH_{ - i}^{j}$ is inversely related to $MNB_{i}^{j}$, increasing in *L,* and decreasing in *Z*. Similarly, as discussed for $MNB_{i}^{j}$, the directional response to treatment conditions resulting from $MH_{ - i}^{j}$ is affected by the critical threshold of first order beliefs of group appropriation of 50 tokens. In sum, while higher values of *Z* increase the magnitude of pecuniary benefits $MNB_{i}^{j}$, higher values of Z decrease the magnitude of the marginal damage associated with appropriation $MH_{ - i}^{j}$.^9^

## Results

### Descriptive overview

Pooling across individuals, Table 2 provides mean individual appropriation and first order beliefs of appropriation of others. On average, aggregate appropriation and forecasts of others’ appropriation decrease as *L* increases and differences in all paired comparisons are statistically significant (see Table A1 in the Electronic Supplementary Material).

**Table 2:** Average appropriation and forecasts of others’ appropriation

	*L0*	*L10*	*L50*	*L90*
*Appropriation*				
Average	13.027	11.793	8.883	8.378
Standard deviation	10.176	9.797	9.534	10.84
*Forecasts*				
Average	11.836	11.247	9.201	8.173
Standard deviation	7.429	6.997	7.201	8.994
*N*	111	111	111	111

**Fig. 2:**
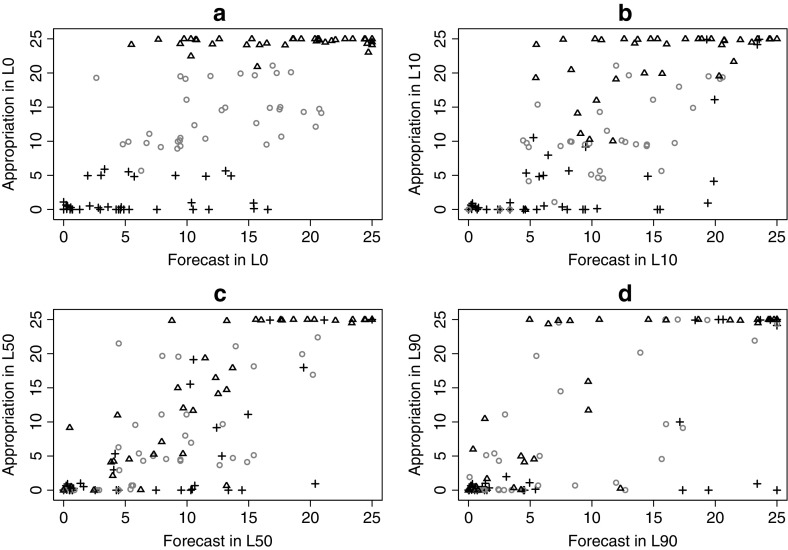
Individual appropriation decisions and forecasts of others’ appropriation

Focusing on heterogeneity in decisions across individuals and across treatments, Fig. 2 provides an illustration of individual appropriation decisions and forecasts in each treatment condition. As expected, in *L0* (where only deterministic degradation exists, and marginal net benefits and harm from appropriation are constant) there is a substantial diversity in subjects’ appropriation decisions, providing evidence of heterogeneity in underlying predispositions toward cooperativeness that are not associated with changes in marginal incentives within the game. The symbols in Fig. 2 are provided to reference the appropriation level of subjects in the *L0* game: a cross refers to low appropriation between 0 and 5 tokens, a triangle refers to high appropriation between 20 and 25 tokens, and a circle subjects refers to intermediate appropriation between 6-19 tokens. Examining the distribution of observations across treatments, one observes many subjects who make appropriation decisions that are quite consistent across games. In addition, there are some subjects who make substantial changes in their appropriation decisions. For example, the triangles in panels c and d near the horizontal axis represent subjects with low levels of appropriation in *L50* and *L90*. These are individuals, however, who appropriated at or near the non-cooperative equilibrium in *L0*. A similar (but opposite) pattern is observed by examining the subjects who had low levels of appropriation in the *L0* game (crosses in panel a), who then made relatively large appropriation decisions in the *L50* and *L90* treatments (crosses on the top range of panels c and d). In addition, we examined to what extent individuals’ expectations of others’ appropriation changed as *L* increased. Interestingly, 61.26% of subjects consistently decreased their expectations, 26.13% consistently increased their expectations, and only 12.61% did not show a consistent change in expectations as *L* increased.

### Individual responses to treatment conditions

The within-subject structure of the data is used by focusing on changes in individuals’ decisions across treatments relative to their decisions in the *L0* game. This allows for testing for treatment effects controlling for the baseline appropriation (subjects’ cooperativeness) where marginal incentives are independent of group appropriation.

Table 3 presents OLS regression results for differences in appropriation between decisions in a given treatment condition and *L0* where the independent variable for each regression is the expected marginal net benefit in each treatment condition E($MNB_{i}^{j}$), *j* = *L10, L50, L90*. This variable is constructed following the functions in Table 1, where expected group appropriation E(Z) by subject *i* in game *j* is the sum of the forecast of *i* of the three other group members plus his/her own appropriation. As shown, for all three paired comparisons, E($MNB_{i}^{j}$) is highly significant.^10^

**Table 3:** Individual appropriation relative to *L0* as a function of expected marginal net benefits

	(1)	(2)	(3)
	*L10*–*L0*	*L50*–*L0*	*L90*–*L0*
E($MNB_{i}^{L10}$)	80.82 (0.000)	–	–
E($MNB_{i}^{L50}$)	–	29.98 (0.000)	–
E($MNB_{i}^{L90}$)	–	–	23.42 (0.000)
Constant	−41.28 (0.000)	−17.11 (0.000)	−12.75 (0.000)
*N*	111	111	111
*R* ^2^	0.114	0.194	0.387

*p* values in parentheses

**Fig. 3:**
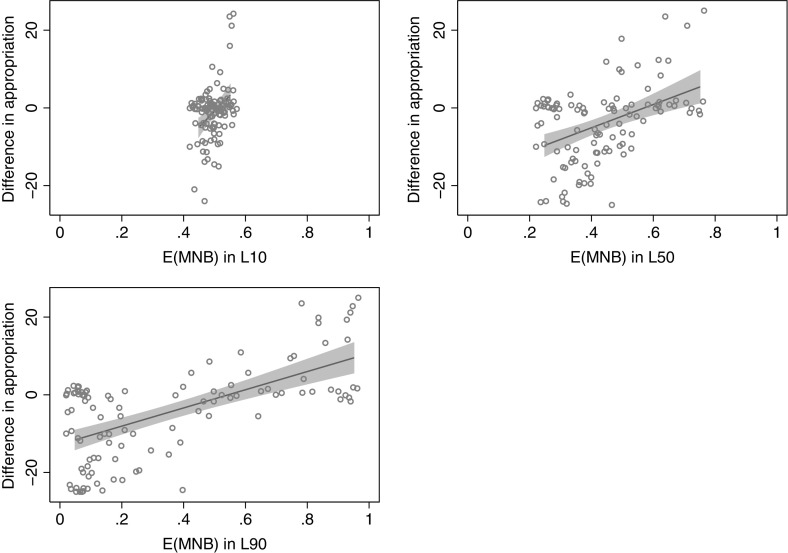
Illustration of results from Table 3

This analysis, however, does not lend itself to a straightforward comparison across treatments on the relevancy of E($MNB_{i}^{j}$) or $E(MH_{ - i}^{j} )$. The reason for this relates to the differences in the range of $MNB_{i}^{j}$ and $MH_{ - i}^{j}$ across treatments. This is illustrated for $MNB_{i}^{j}$ in Fig. 3, which displays scatter plots of individual appropriation decisions, expected marginal net benefits, and regression lines with 95% confidence intervals of the estimated OLS models. In particular, note the differences in the range of $MNB_{i}^{j}$ displayed on the horizontal axis for the different treatment conditions.

As a resolution to the comparability issue described above, Table 4 presents the results from an alternative OLS analysis where the explanatory variable is the expected total group appropriation, E(Z).^11^ As shown, the coefficient for E(Z) is positive and highly significant in all treatment conditions. Further, comparisons across treatments show that both the constant term and the coefficient of E(Z) significantly increase in absolute magnitude as *L* increases.^12^ Thus, in addition to the result that own appropriation increases with forecasts of group appropriation, treatment effects show that the magnitude of that response is stronger with increases in the probabilistic loss parameter *L*. This result is consistent with the discussion in Sect. 3 on responses to changes in the magnitude of the loss parameter based on subjects responding to changes in expected marginal net benefits or to changes in expected harm to others.

**Table 4:** Individual appropriation relative to *L0* as a function of expected group appropriation

	(1)	(2)	(3)
	*L10*–*L0*	*L50*–*L0*	*L90*–*L0*
E(Z)	0.081 (0.001)	0.150 (0.000)	0.211 (0.000)
Constant	−4.914 (0.000)	−9.613 (0.000)	−11.58 (0.000)
*N*	111	111	111
*R* ^2^	0.114	0.194	0.387

*p* values in parentheses

A natural extension of the analysis in Table 4 is to examine whether the responses to changes in expectations of group appropriation are symmetric for subjects whose forecasts of group appropriation are above and below the threshold of 50 tokens referenced in Fig. 1, where expected marginal incentives in *L10*, *L50*, and *L90* equal that of *L0*. We use this objective reference point to define what we refer to as pessimistic and optimistic subjects regarding group cooperation. Given the appropriation frame used in this study, pessimistic subjects are those expecting high appropriation levels (above 50) and optimistic subjects are those expecting low appropriation levels (below or equal to 50). Table 5 presents OLS results that parallel the approach presented in Table 4, except that the analysis is conducted separately for subjects with expected group appropriation in a given treatment above 50 tokens (columns 1-3) and for those with expectations below or equal to 50 tokens (columns 4-6). For this analysis, the variable $E\left( Z \right)$ is transformed to $E\left( {Z - 50} \right)$. Thus, the variable $E\left( {Z - 50} \right)$ takes on values from 1 to 50 for the group of subjects with expectations of total group appropriation above the threshold, and −50 to 0 for those with expectations below the threshold. It follows that the estimated constant term (in all columns) provides information on the appropriation levels relative to the threshold of 50 tokens. Given that marginal net benefits to appropriate are identical at the threshold, we would expect none of the intercept terms to be significantly different from zero if subjects responded exclusively to expected $MNB_{i}^{j}$.

**Table 5:** Individual appropriation relative to *L0* as a function of expected group appropriation: pessimistic and optimistic subjects

	“Pessimistic” expectations above 50			“Optimistic” expectations below 50^a^		
	(1)	(2)	(3)	(4)	(5)	(6)
	*L10*–*L0*	*L50*–*L0*	*L90*–*L0*	*L10*–*L0*	*L50*–*L0*	*L90*–*L0*
E(Z-50)	0.142 (0.046)	0.189 (0.044)	0.201 (0.038)	−0.0379 (0.420)	0.0704 (0.261)	0.113 (0.149)
Constant	−1.755 (0.391)	−2.229 (0.416)	−0.0499 (0.988)	−4.182 (0.003)	−4.737 (0.025)	−5.115 (0.114)
*N*	44	31	34	67	80	77
*R* ^2^	0.091	0.133	0.128	0.010	0.016	0.028

^a^Includes subject with expectations exactly at 50 tokens

*p* values in parentheses

Pessimistic subjects systematically respond to changes in first order beliefs (significant coefficient for $E\left( {Z - 50} \right)$) while optimistic subjects do not. Moreover, the constant terms for the optimistic subjects are negative and statistically significant. The significantly negative intercept terms shown in columns 4 and 5 for optimistic subjects indicates that, despite marginal incentives being equal at the threshold there is a significant downward shift in appropriation for subjects in the *L10* and *L50* treatments as compared to the benchmark *L0* condition.

To gain further insight into this result, we examine whether pessimistic and optimistic subjects differ in their underlying cooperativeness in game *L0* and in Part D of the experiment, where they make donation decisions to charities.^13^ We find that the subjects we classify as optimistic make appropriation decisions in *L0* that are more cooperative than those we classify as pessimistic. These differences are statistically significant for treatments *L10* and *L50,* but not so for *L90*. Similarly, we find that optimistic subjects donate more to the charities than pessimistic subjects. In these comparisons, however, the mean differences are statistically significant only for *L50* (see Tables A4 and A5 in the Electronic Supplementary Material).

## Discussion of results and conclusions

This study examines how subjects respond to changes in the vulnerability of a shared resource to appropriation pressures. Experimentally we vary the magnitude of a probabilistic loss of a group fund, where the probability of occurrence of the loss increases in appropriation. Thus, this study adds to the experimental literature that examines subjects’ responses to endogenous probabilistic losses in social dilemmas (Walker and Gardner 1992; Dickinson 1998; Gangadharan and Nemes 2009; Blanco et al. 2016b). We contribute to this literature by providing the first results on the quantitative response to variations in the *magnitude* of endogenous losses rather than just to the *existence* of the endogenous component.

We find that average group cooperation increases as the loss parameter (*L*) increases; with important heterogeneities in individual behavior. Controlling for individual decisions in the benchmark game without probabilistic losses, we observe a behavioral difference between those subjects who forecast lower levels of group cooperation versus those who forecast higher levels of group cooperation. In particular, subjects who are pessimistic regarding others’ appropriation appropriate at higher levels on average (84% of their appropriation capacity), and respond systematically and significantly to changes in their expectations of others’ appropriation. Subjects who are optimistic about other’s appropriation appropriate at lower levels on average (16% of their appropriation capacity). However, the latter group does not make appropriation decisions that are as systematically linked to changes in expectations of group appropriation.

More generally, these novel results add to the emergent literature that explores individual differences in the responses to marginal incentives and reciprocity. In particular, we show the relevance of threshold expectations on group cooperation in defining subjects’ responsiveness (or lack of it) to others’ behavior. These findings extend the results reported in Goeree et al. (2002), for provision decisions in a public good setting with deterministic marginal benefits. In that study, the authors observe, on average, a positive relationship between public good contributions and the internal return to the contributor, as well as the return to other group members. However, the effect of the internal return is larger and more systematic, as their model estimates of individual’s altruism toward others suggests considerable heterogeneity in responses.

What are possible explanations for the behavioral differences we observe between optimistic and pessimistic subjects in this study? Suppose subjects’ behavior focuses primarily on individual expected marginal incentives. In the context of our decision setting, pessimistic expectations of higher group appropriation imply subjects perceive, relative to the benchmark condition, a higher expected marginal private return from appropriation and a lower expected marginal harm to the group. In this sense, if our subjects’ individual response to incentives is consistent with that observed in Goeree et al. (2002), across the group of pessimistic subjects, the increase in appropriation relative to their baseline appropriation can be expected to make appropriation decisions that correlate more systematically with their expectations of others’ appropriation. And, this is in fact what we observe for the pessimistic subjects.

However, optimistic expectations of lower group appropriation imply subjects perceive, relative to the benchmark condition, a lower expected marginal private return from appropriation and a higher expected marginal harm to the group. In line with the results from Goeree et al. (2002), we find that the optimistic subjects lower appropriation overall. However, in terms of statistical significance, they do not respond as systematically to changes in expected marginal incentives. One could alternatively suppose that optimistic subjects’ decisions are influenced more strongly by additional motives such as warm-glow from the act of cooperating or fairness heuristics. These additional motivations could lead to decisions by optimistic subjects that are less sensitive to changes in pecuniary incentives inferred from changes in expectations of others’ behavior. In the context of our experiment, such motivations would be compatible with the evidence that the optimistic group made more cooperative decisions in the benchmark game, which is what we observe.

In summary, in very different decision settings, one where marginal incentives are deterministic and one where they are endogenous and dependent on expectations of others’ behavior, both Goeree et al. (2002) and this study find evidence that decisions are consistent with a more systematic response to changes in private marginal incentives relative to the impact on other group members. In our study, this heterogeneity in behavior is linked to threshold expectations of whether subjects are more (or less) pessimistic about the actions of other group members.

The policy implications of these results can be illustrated by the motivating example of the maintenance of ecosystem services provided in the introduction. As this study demonstrates, individuals may have heterogeneous responses to the potential of increasingly severe endogenous destruction of a resource. While some individuals might be willing to make necessary sacrifices in resource use by limiting their appropriation (despite pecuniary incentives), others might perceive conservation objectives to be unrealistic (or unfeasible) and thus engage in highly extractive strategies leading to a race-to-the-bottom. Which of these strategies individuals undertake might be (at least partially) influenced by their first order beliefs of others’ behavior.

## Electronic supplementary material

Below is the link to the electronic supplementary material.

Supplementary material 1 (DOCX 99kb)

## Footnotes

1 Earlier studies addressing decision environments in which subjects’ decisions are framed as providing a public good or preventing a public bad include by Andreoni (1995), Sonnemans et al. (1998), Dufwenberg et al. (2011), Cox et al. (2013), Hoyer et al. (2014) and Khadjavi and Lange (2015). Unlike these studies, this study is not designed to investigate the effect of alternative frames of the social dilemma.

2 The game studied here captures the essence of probabilistic degradation externalities, while abstracting away from production externalities normally associated with appropriation in common-pool resource settings. Extensive field and experimental research has focused on *production externalities* (e.g., Agrawal 2001; Baland and Platteau 1996; Ostrom 1990; Ostrom et al. 1992, 1994; Wade 1988). Production externalities are generally viewed as situations where appropriation increases the cost per unit of appropriation or increases the effort required per unit appropriated for all users. By focusing primarily on production externalities, this literature has largely neglected the relevance of ecosystem services provided by the natural resources from which appropriation occurs.

3 In this way, the results reported herein complement the findings in Goeree et al. (2002). These authors examine provision of a public good, where contributions create an “internal return” to the contributor that can differ from the “external return” to other group members with deterministic parameters. A greater internal return lowers the cost of contributing while a higher external return increases the benefit to other group members.

4 The subject population for this study was students from the University of Innsbruck, Austria, whereas the subject population for Blanco et al. (2016b) was from the Pontificia Universidad Javeriana, Colombia. The results reported herein are based solely on the data from the University of Innsbruck.

5 All values of *L* were presented to subjects as percentages.

6 The number of subjects is not divisible by four due to the fact that one participant left one of the sessions before it was completed. The session continued with each participant making their decisions, without feed-back. Because of the one-shot nature of the decision setting, the formation of groups was only implemented in order to calculate payments, after all decisions were final. There was no group feed-back or identification of groups during the session. For the group with three members, the average appropriation of the three group members was used for the 4th member to compute payments. At no point during the session did any of the participants raise a question or concern about this one subject leaving the session.

7 Earnings in Part B, C and D were denoted in Euros. The exchange rate at the time was US$1.36 per Euro. On average subjects total earnings were 8.5 Euros (US$11.47) during the experiment which lasted approximately 60 min. The results for the risk aversion task are not reported for brevity, as it was not found to be significantly correlated with game decisions, potentially due to the small variability in the choices made.

8 Except in the benchmark game, where *L* = 0 and $MNB_{i}^{j}$ equals 0.5, independent of expected group appropriation.

9 Disentangling how subjects respond to the tension between private marginal benefits and harm to other group members is beyond the scope of this study. Instead, we focus on addressing the scope and heterogeneity in responses to changes in the magnitude of changes in *L*.

10 Using $E(MH_{ - i}^{j} )$ as the explanatory variable does not alter the results. See Table A2 in the Electronic Supplementary Material.

11 Using forecast of others leads to virtually the same result exept for the absolute magnitude of the coefficients of the independent variable. These additional results are reported in Table A3 in the Electronic Supplementary Material.

12
*p* values for corresponding Wald tests on changes in the constant term: *L10* versus *L50* (0.008), *L50* versus *L90* (0.000), *L10* versus *L90* (0.000). *p* values for corresponding Wald tests on changes in the coefficient: *L10* versus *L50* (0.000), *L50* versus *L90* (0.003), *L10* versus *L90* (0.000).

13 By construction, the composition of subjects in the optimistic groups can vary across games. However, the overall number of subjects in the optimistic group holds relatively steady, with an average of 75 subjects. Further, there is fairly strong evidence that the composition of the optimistic groups do not change dramatically across games in the sense that 64 subjects forecast total group appropriation of 50 or below in 3 out of 4, or 4 out of 4 games. There was also no evidence of a gender bias in the composition. The percentage of females in the optimistic groups holds relatively stable at a level that is almost identical to the percentage of females in the subject pool.
