# Supplementary material for: Externalities in appropriation: responses to probabilistic losses
Source: Exp Econ. 2017 Feb 10;20(4):793–808. doi: 10.1007/s10683-017-9511-x (PMC5665978; doi:10.1007/s10683-017-9511-x)
Supplement: Supplementary file 1 — Supplementary material 1 (DOCX 99kb) [file 10683_2017_9511_MOESM1_ESM.docx]

**Electronic Supplementary Material**

**Externalities in appropriation: Responses to probabilistic losses**

**by**

**Esther Blanco, Tobias Haller, and James M. Walker.**

Table of Contents:

Section I: Additional analyses 2

Section II: Instructions 4

# Section I: Additional analyses

**Table A1.** Mean paired differences in appropriation and forecasts across treatment conditions

| Appropriation | | | | | | |
| --- | --- | --- | --- | --- | --- | --- |
|  | *L10-L0* | *L50-L0* | *L90-L0* | *L50-L10* | *L90-L10* | *L90-L50* |
| Mean difference | -1.234 | -4.144 | -4.649 | -2.910 | -3.414 | -0.505 |
| Wilcoxon signed rank test | z=-2.802 (0.005) | z=-4.535 (0.000) | z=-3.913 (0.000) | z=-4.407 (0.000) | z=-3.992 (0.000) | z=-1.954 (0.051) |
| Forecasts | | | | | | |
|  | *L10-L0* | *L50-L0* | *L90-L0* | *L50-L10* | *L90-L10* | *L90-L50* |
| Mean difference | -0.589 | -2.635 | -3.663 | -2.047 | -3.074 | -1.027 |
| Wilcoxon signed rank test | z=-2.156 (0.031) | z=-3.668 (0.000) | z=-3.721 (0.000) | z=-4.402 (0.000) | z=-4.044 (0.000) | z=-2.945 (0.003) |
| *N* | 111 | 111 | 111 | 111 | 111 | 111 |

p-values for Wilcoxon signed rank tests in parentheses

Tests of statistical significance are based on Wilcoxon signed rang tests rather than t-tests as the normality condition is not satisfied for all differences in appropriation and forecasts. The results from t-tests are consistent with these results except for the appropriation *L90-L50* t-test: t=-0.826 (0.411), and the forecast *L10-L0* t-test: t=-1.002 (0.318).

**Table A2**. Individual appropriation relative to *L0* as a function of expected marginal harm to others.

|  | (1) | (2) | (3) |
| --- | --- | --- | --- |
|  | *L10-L0* | *L50-L0* | *L90-L0* |
| E(${MH}_{i}^{L10}$) | 26.94 (0.000) | - | - |
| E(${MH}_{i}^{L50}$) | - | 9.994 (0.000) | - |
| E(${MH}_{i}^{L90}$) | - | - | 7.806 (0.000) |
|  |  |  |  |
| constant | 39.54 (0.000) | 12.87 (0.000) | 10.66 (0.000) |
| *N* | 111 | 111 | 111 |
| *R*^2^ | 0.114 | 0.194 | 0.387 |

*p*-values in parentheses

**Table A3.** Individual appropriation relative to *L0* as a function of the forecast of other group members’ average appropriation.

|  | (1) | (2) | (3) |
| --- | --- | --- | --- |
|  | *L10-L0* | *L50-L0* | *L90-L0* |
| Forecast of others’ appropriation | 0.322 (0.000) | 0.557 (0.000) | 0.782 (0.000) |
|  |  |  |  |
| constant | -4.854 (0.000) | -9.267 (0.000) | -11.04 (0.000) |
| *N* | 111 | 111 | 111 |
| *R*^2^ | 0.106 | 0.159 | 0.336 |

*p*-values in parentheses

**Table A4.** Difference in appropriation in *L0* depending on first order beliefs of total group appropriation

| **EXPECTATIONS**  **ABOVE 50** | | | | | |
| --- | --- | --- | --- | --- | --- |
|  |  | *L0*  n=53 | *L10*  n=44 | *L50*  n=31 | *L90*  n=34 |
| **EXPECTATIONS BELOW 50** | *L0*  n=58 | -16.522  z=-8.443  (0.000) | - | - | - |
|  | *L10*  n=67 | - | -10.046  z=-5.155  (0.000) | - | - |
|  | *L50*  n=80 | - | - | -7.840  z=-3.733  (0.000) | - |
|  | *L90*  n=77 | - | - | - | -0.809  z=-0.639  (0.523) |

p-values for Wilcoxon signed rank tests in parentheses

**Table A5**. Difference in donation decisions to charities depending on first order beliefs of total group appropriation

| **EXPECTATIONS**  **ABOVE 50** | | | | | |
| --- | --- | --- | --- | --- | --- |
|  |  | *L0*  n=53 | *L10*  n=44 | *L50*  n=31 | *L90*  n=34 |
| **EXPECTATIONS BELOW 50** | *L0*  n=58 | -0.131  z=-0.476  (0.634) | - | - | - |
|  | *L10*  n=67 | - | -0.267  z=-1.252  (0.211) | - | - |
|  | *L50*  n=80 | - | - | -0.422  z=-1.782  (0.075) | - |
|  | *L90*  n=77 | - | - | - | -0.150  z=-0.631  (0.528) |

p-values for Wilcoxon signed rank tests in parentheses

# Section II: Instructions

*The instructions were in German. Below we present an English translation.*

**General Instructions**

**WELCOME**

This is an experiment on the economics of decision making. You will have the chance to earn money based on your decisions in this experiment. It is extremely important that you put away all materials including external reading material and turn off your cell phones and any other electronic devices. If you have a question, please raise your hand and I will come by and answer your question privately. No talking is permitted. Please read the instructions carefully, as your decisions and the decisions of others in the experiment will affect your final earnings.

When you entered the room you received a **participant number**. Please write this number in all of your decision sheets as we distribute them to you. Please do not write in these instructions.

Today’s experiment is comprised of four parts, Part A, Part B, Part C, and Part D. Your earnings from the four parts are calculated independently for each part. Your total earnings from the experiment will be the sum of your payments in parts A, B, C, and D. At the end we will ask you to answer a short survey.

The following instructions are for part A. Prior to the start of the other parts, additional instructions will be given.

**PART A - Experiment Instructions**

In this part of the experiment, you will make choices in 4 independent decision situations.

- You will receive specific instructions for each decision situation.
- Before making decisions for each decision situation, you will answer a short quiz designed to check your understanding of that decision situation. After all participants finish each quiz the monitor will collect the quizzes and I will provide the solutions in public and answer questions privately.
- At any point during decision-making, you will have the opportunity to review and (if you wish) change any of the choices that you have already made. After all participants have had time to finalize their decisions, the monitor will announce the end of Part A of the experiment, after which no one will be allowed to change their decisions from this part.
- Groups of 4 persons have been randomly created based on participant numbers.
- Your cash earnings will depend on your decisions and the decisions of the other three participants with whom you are grouped.
- At the end of the experiment, after the four parts of the experiment and the survey are completed, we will **randomly pick one of the 4 decision situations in Part A for computing your cash earnings for this part**. The draw will be made by picking a card out of a shuffled deck of cards numbered from 1 to 4. The drawing will be made in public at the front of the room. The decision situation chosen based on the card drawn will be the same for all groups.
- Your decisions and earnings are your private information. These decisions will be recorded only by your participant number and **not** your name.
- All decision situations are described in **Experimental Currency Units (ECUs)**. At the end of the experiment you will be paid in cash at a rate of **30 cents** for every ECU you earn. You are free to leave at any point during the experiment, however if you decide to leave before the end of the experiment you will not be paid.
- The experiment will last approximately one to one and a half hours.

**DECISION SITUATION 1**

In today’s experiment, you will have an *Individual Fund* and your group of four will have a *Group Fund*.

**STARTING BALANCES:** Each **group of four** begins with 100 tokens placed in their initial *Group Fund*. Each token in the initial *Group Fund* is worth **2 ECUs**. Thus, each group begins with an initial *Group Fund* worth **200 ECUs**. Each **participant** begins with 0 tokens placed in his/her initial *Individual Fund*.

**DECISION TASK:** Each participant will decide privately whether or not to move tokens from the initial *Group Fund* to his/her own *Individual Fund*.

Each participant can move up to a maximum of 25 tokens from the initial *Group Fund* to his/her own *Individual Fund*. **Each token that a participant moves from the initial *Group Fund* increases the value of his/her own *Individual Fund* by 1 ECU. However, each token moved from the initial *Group Fund* reduces the value of the final *Group Fund* by 2 ECUs for his/her group**. Each participant’s decision must be in whole tokens from 0 up to a maximum of 25 (0,1,2, ..., 24 or 25).

**EARNINGS: In each group of four, a participant’s earnings will be the sum of the value of that participant’s *Individual Fund* plus a fourth (¼) of the value of the final *Group Fund*.**

In summary, every token a participant moves to his Individual Fund increases his earnings by 1 ECU and reduces the value of the Group Fund by 2 ECUs; this reduces his earnings and the earnings of every other participant in his group by 0.5 ECUs.

If there are any questions, please raise your hand and we will come to you and answer them privately.

*Examples*

Let’s go through four examples.

Example 1: Suppose you did not move any tokens to your Individual Fund. Also suppose that none of the other participants moved any tokens to their Individual Funds.

With these assumptions, you would earn **0 ECUs** in your Individual Fund

*Plus* you would earn **¼** of the final Group Fund, which would hold 100 tokens that are worth 2 ECUs each

= **¼** of 200 ECUs = **50 ECUs**

**YOUR TOTAL EARNINGS WOULD BE 50 ECUs**

**Since all other participants are making the same decision as you, they would also earn a total of 50 ECUs each.**

Example 2: Suppose that every participant in your group moved 5 tokens to their Individual Fund, for a total of 20 tokens removed. In this case, you would earn

**5 ECUs** in your Individual Fund

*Plus* you would earn **¼** of the final Group Fund, which would hold 80 tokens (100-20 tokens) that are worth 2 ECUs each

= **¼** of 160 ECUs = **40 ECUs**

**YOUR TOTAL EARNINGS WOULD BE 45 ECUs**

**In this example, since all other participants in your group are making the same decisions as you, they would also earn a total of 45 ECUs each.**

Example 3: Suppose you moved 20 tokens to your Individual Fund and the others in your group moved 0 tokens to their Individual Fund. In this case you would earn

**20 ECUs** in your Individual Fund

*Plus* you would earn **¼** of the final Group Fund, which would hold 80 tokens (100-20 tokens) that are worth 2 ECUs each

= **¼** of 160 ECUs = **40 ECUs**

**YOUR TOTAL EARNINGS WOULD BE 60 ECUs**

**In this example, since none of the other participants moved any tokens to their Individual Fund, they would earn 0 ECUs from their Individual Fund. Plus, like you, they would receive 40 ECUs from the Group Fund. Thus, their total earnings would be 40 ECUs each.**

Example 4: Lastly, suppose that every participant in your group moved all 25 tokens to their Individual Funds. In this case, you and each of the other group members would earn

**25 ECUs** in your Individual Funds

*Plus* each of you would have no earnings from the Group Fund because all 100 tokens were removed

**Thus, the total earnings for each of you would be 25 ECUs.**

**Please complete quiz 1 now.**

**Quiz 1: Participant number ___________**

1.1. In Decision Situation 1, the starting value of your *Individual Fund* is ____ ECUs.

1.2. In Decision Situation 1, the starting value of the initial *Group Fund* is _____ ECUs.

1.3. In Decision Situation 1, each token you move from the initial *Group Fund* increases the value of your *Individual Fund* by ____ ECUs and reduces the value of the final *Group Fund* by _____ ECUs.

**DECISION SITUATION 2**

Decision Situation 2 is the same as Decision Situation 1, except for the following change: **For each token removed from the initial *Group Fund* by a member of your group, in addition to the reduction of 2 ECUs, there is a 1% chance that the value of the final *Group Fund* is reduced by one-half, that is by 50%.**

Otherwise, all other aspects are the same as in Decision Situation 1.

After all decisions are made, **if Decision 2 is randomly drawn for determining cash earnings**, the following procedure will be followed.

- A deck of cards numbered 1-100 will be displayed and shuffled. I will draw one card from the deck of cards. The drawing will be made in public, at the front of the room. This card will be used for all groups.
- **For each group of four**, **if the card drawn is greater than the number of tokens removed from the initial *Group Fund*,** then the value of the final *Group Fund* will not be reduced. **If the card drawn is less than or equal to the number of tokens removed from the initial *Group Fund***, the value of the final *Group Fund* will be reduced by half (½) of its ending value.

**EARNINGS: In each group of four, an individual’s earnings will be the sum of the value of that participant’s *Individual Fund* plus a fourth (¼) of the value of the final** ***Group Fund* for his/her group.**

In summary, every token a participant moves to his *Individual Fund* increases his earnings by 1 ECU and reduces the value of the Group Fund by 2 ECUs. But in addition, for every token moved there is a 1% probability that the value of the final *Group Fund* is reduced by 50%.

If there are any questions, please raise your hand and we will come to you and answer them privately.

*Examples*

Let’s go through four examples.

Example 1: Suppose you did not move any tokens to your Individual Fund. Also suppose that none of the other participants moved any tokens to their Individual Funds.

With these assumptions, you would earn **0 ECUs** in your Individual Fund

*Plus since there is a 0% probability of reduction of the final Group Fund value*

you would earn **¼** of the final Group Fund, which would hold 100 tokens that are worth 2 ECUs each

= **¼** of 200 ECUs = **50 ECUs**

**YOUR TOTAL EARNINGS WOULD BE 50 ECUs**

**In this example, since all other participants are making the same decision as you, they would also earn a total of 50 ECUs each.**

Example 2: Suppose that every participant in your group moved 5 tokens to their Individual Fund, for a total of 20 tokens removed. In this case, you would earn

**5 ECUs** in your Individual Fund

Plus your earnings from the Group Fund would be calculated as follows

*Because a total of 20 tokens were removed from the Group Fund, this means there is a 20% probability that the final Group Fund will be reduced by 50% and an 80% probability that the final Group Fund will not be reduced.*

***Thus, if the card drawn was larger than 20 (which has an 80% probability)*** ***the value of the final Group Fund would not be reduced, so you would earn:***

**¼** of the final Group Fund, which would hold 80 tokens (100-20 tokens) that are worth 2 ECUs each = **¼** of 160 ECUs = **40 ECUs**

***If the card drawn was equal to or smaller than 20 (which has a 20% probability) the value of the final Group Fund would be reduced by 50%, so you would earn:***

**¼** of 160 ECUs - **50%** x 160 ECUs (80 ECUs) = ¼ of 80 ECUs (160 - 80) = **20 ECUs**

**YOUR TOTAL EARNINGS WOULD BE DETERMINED AS FOLLOWS**

$$\boldsymbol{=}\left\{ \begin{matrix} \boldsymbol{if no reduction, 45 ECUs = 5 from your Individual Fund+ 40 from the Group Fund} \\ \boldsymbol{if reduction, 25 ECUs = 5 from your Individual Fund+ 20 from the Group Fund} \end{matrix} \right.$$

**In this example, since all of other participants are making the same decision as you, they would also earn the same total earnings as you.**

Example 3: Suppose you moved 20 tokens to your Individual Fund and the others in your group moved 0 tokens to their Individual Fund. In this case you would earn

**20 ECUs** in your Individual Fund

Plus your earnings from the Group Fund would be calculated as follows

*Because a total of 20 tokens were removed from the Group Fund, this means there is a 20% probability that the final Group Fund will be reduced by 50% and an 80% probability that the final Group Fund will not be reduced.*

***Thus, if the card drawn was larger than 20 (which has an 80% probability)*** ***the value of the final Group Fund would not be reduced, so you would earn:***

**¼** of the final Group Fund, which would hold 80 tokens (100-20 tokens) that are worth 2 ECUs each = **¼** of 160 ECUs = **40 ECUs**

***If the card drawn was equal to or smaller than 20 (which has a 20% probability) the value of the final Group Fund would be reduced by 50%, so you would earn:***

**¼** of 160 - **50%** x 160 ECUs (80 ECUs) = ¼ of 80 ECUs (160 - 80)= **20 ECUs**

**YOUR TOTAL EARNINGS WOULD BE DETERMINED AS FOLLOWS**

$$\boldsymbol{=}\left\{ \begin{matrix} \boldsymbol{if no reduction, 60 ECUs = 20 from your Individual Fund+ 40 from the Group Fund} \\ \boldsymbol{if reduction, 40 ECUs = 20 from your Individual Fund+ 20 from the Group Fund} \end{matrix} \right.$$

**In this example, since none of the other participants moved any tokens to their Individual Funds, they would earn 0 ECUs from their Individual Funds. Plus, they would receive the same earnings as you from the Group Fund.**

Example 4: Lastly, suppose that every participant in your group moved all 25 tokens to their Individual Funds. In this case, you and each of the other group members would earn

**25 ECUs** in your Individual Funds

*Plus* each of you would have no earnings from the Group Fund because all 100 tokens were removed*.*

**Thus, the total earnings for each of you would be 25 ECUs.**

**Please complete quiz 2 now.**

**Quiz 2: Participant number ___________**

2.1. In Decision Situation 2 the starting value of your *Individual Fund* is ____ ECUs.

2.2. In Decision Situation 2, the starting value of the initial *Group Fund* is _____ ECUs.

2.3. In Decision Situation 2, each token you move from the initial *Group Fund* increases the value of your *Individual Fund* by ____ ECUs and reduces the value of the final *Group Fund* by _____ ECUs. In addition, each token a group member removes from the initial *Group Fund* increases by 1% the probability that the final *Group Fund* will reduce **______%**  of its value.

**DECISION SITUATION 3**

Decision Situation 3 is the same as Decision Situation 2, except for the following change: **For each token removed from the initial *Group Fund* by a member of your group, in addition to the reduction of 2 ECUs, there is a 1% chance that the value of the final *Group Fund* is reduced by one-tenth, that is by 10%.**

As in decision situation 2, I will randomly pick a card to determine whether or not the value of the final *Group Fund* will be reduced, in this case by 10% of its ending value.

In summary, every token a participant moves to his Individual Fund increases his earnings by 1 ECU and reduces the value of the Group Fund by 2 ECUs. But in addition, for every token moved there is a 1% probability that the value of the final Group Fund is reduced by 10%.

If there are any questions, please raise your hand and we will come to you and answer them privately.

*Examples*

Examples 1 and 4 are identical to the examples in Decision Situation 2. Let's see what happens with examples 2 and 3.

Example 2: Suppose that every participant in your group moved 5 tokens to their Individual Fund, for a total of 20 tokens removed. In this case, you would earn

**5 ECUs** in your Individual Fund

Plus your earnings from the Group Fund would be calculated as follows

*Because a total of 20 tokens were removed from the Group Fund, this means there is a 20% probability that the final Group Fund will be reduced by 10% and an 80% probability that the final Group Fund will not be reduced.*

***Thus, if the card drawn was larger than 20 (which has an 80% probability)*** ***the value of the final Group Fund would not be reduced, so you would earn:***

**¼** of the final Group Fund, which would hold 80 tokens (100-20 tokens) that are worth 2 ECUs each = **¼** of 160 ECUs = **40 ECUs**

***If the card drawn was equal to or smaller than 20 (which has a 20% probability) the value of the final Group Fund would be reduced by 10%, so you would earn:***

**¼** of 160 ECUs - **10%** x 160 ECUs (16 ECUS)= ¼ of 144 ECUs (160-16)=  **36 ECUs**

**YOUR TOTAL EARNINGS WOULD BE DETERMINED AS FOLLOWS**

$$\boldsymbol{=}\left\{ \begin{matrix} \boldsymbol{if no reduction, 45 ECUs = 5 from your Individual Fund+ 40 from the Group Fund} \\ \boldsymbol{if reduction, 41 ECUs = 5 from your Individual Fund+ 36 from the Group Fund} \end{matrix} \right.$$

**In this example, since all of other participants are making the same decision as you, they would also earn the same total earnings as you.**

Example 3: Suppose you moved 20 tokens to your Individual Fund. Also suppose that none of the other participants moved any tokens to their Individual Funds. With these assumptions, you would earn

**20 ECUs** in your Individual Fund

*Plus* your earnings from the Group Fund would be calculated as follows

*Because a total of 20 tokens were removed from the Group Fund, this means there is a 20% probability that the final Group Fund will be reduced by 10% and an 80% probability that the final Group Fund will not be reduced.*

***Thus, if the card drawn was larger than 20 (which has an 80% probability)*** ***the value of the final Group Fund would not be reduced, so you would earn:***

**¼** of the final Group Fund, which would hold 80 tokens (100-20 tokens) that are worth 2 ECUs each = **¼** of 160 ECUS = **40 ECUs**

***If the card drawn is equal to or smaller than 20 (which has a 20% probability) the value of the final Group Fund would be reduced by 10%, so you would earn:***

**¼** of 160 ECUs **- 10%** x 160 ECUs (16 ECUs)= ¼ of 144 ECUs (160-16) = **36 ECUs**

**YOUR TOTAL EARNINGS WOULD BE DETERMINED AS FOLLOWS**

$$\left\{ \begin{matrix} \boldsymbol{if no reduction, 60 ECUs = 20}\boldsymbol{from your Individual Fund}\boldsymbol{+ 40}\boldsymbol{from the Group Fund} \\ \boldsymbol{if reduction, 56 ECUs = 20}\boldsymbol{from your Individual Fund}\boldsymbol{+ 36}\boldsymbol{from the Group Fund} \end{matrix} \right.$$

**Since none of the other participants moved any tokens to their Individual Funds, they would earn 0 ECUs from their Individual Funds. Plus, they would receive the same earnings as you from the Group Fund.**

**Please complete quiz 3 now.**

**Quiz 3: Participant number ___________**

3.1. In Decision Situation 3 the starting value of your *Individual Fund* is ____ ECUs.

3.2. In Decision Situation 3, the starting value of the initial *Group Fund* is _____ ECUs.

3.3. In Decision Situation 3, each token you move from the initial *Group Fund* increases the value of your *Individual Fund* by ____ ECUs and reduces the value of the final *Group Fund* by _____ ECUs. In addition, each token a group member removes from the initial *Group Fund* increases by 1% the probability that the final *Group Fund* will reduce **______%** of its value.

**DECISION SITUATION 4**

Decision Situation 4 is the same as Decision Situations 2 and 3, except for the following change: **For each token removed from the initial *Group Fund* by a member of your group, in addition to the reduction of 2 ECUs, there is a 1% chance that the value of the final *Group Fund* is reduced by nine-tenths, that is by 90%.**

As in decision situations 2 and 3, I will randomly pick a card to determine whether or not the value of the final *Group Fund* will be reduced, in this case by 90% of its ending value.

In summary, every token a participant moves to his Individual Fund increases his earnings by 1 ECU and reduces the value of the Group Fund by 2 ECUs. But in addition, for every token moved there is a 1% probability that the value of the final Group Fund is reduced by 90%.

If there are any questions, please raise your hand and we will come to you and answer them privately.

*Examples*

Examples 1 and 4 from above are identical to the examples in Decision Situation 3. Let's see what happens with examples 2 and 3.

Example 2: Suppose that every participant in your group moved 5 tokens to their Individual Fund, for a total of 20 tokens removed. In this case, you would earn

**5 ECUs** in your Individual Fund

Plus your earnings from the Group Fund would be calculated as follows

*Because a total of 20 tokens were removed from the Group Fund, this means there is a 20% probability that the final Group Fund will be reduced by 90% and an 80% probability that the final Group Fund will not be reduced.*

***Thus, if the card drawn was larger than 20 (which has an 80% probability)*** ***the value of the final Group Fund would not be reduced, so you would earn:***

**¼** of the final Group Fund, which would hold 80 tokens (100-20 tokens) that are worth 2 ECUs each = **¼** of 160 ECUs = **40 ECUs**

***If the card drawn was equal to or smaller than 20 (which has a 20% probability) the value of the final Group Fund would be reduced by 90%, so you would earn:***

**¼** of 160 - **90%** x 160 ECUs = ¼ of 16 ECUs (160 - 144)= **4 ECUs**

**YOUR TOTAL EARNINGS WOULD BE DETERMINED AS FOLLOWS**

$$\boldsymbol{=}\left\{ \begin{matrix} \boldsymbol{if no reduction, 45 ECUs = 5 from your Individual Fund+ 40 from the Group Fund} \\ \boldsymbol{if reduction, 9 ECUs = 5 from your Individual Fund+ 4 from the Group Fund} \end{matrix} \right.$$

**In this example, since all of other participants are making the same decision as you, they would also earn the same total earnings as you.**

Example 3: Suppose you moved 20 tokens to your Individual Fund. Also suppose that none of the other participants moved any tokens to their Individual Funds. With these assumptions, you would earn

**20 ECUs** from your Individual Fund

*Plus* your earnings from the Group Fund would be calculated as follows

*Because a total of 20 tokens were removed from the Group Fund, this means there is a 20% probability that the final Group Fund will be reduced by 90% and an 80% probability that the final Group Fund will not be reduced.*

***If the card drawn is bigger than 20 (which has an 80% probability)*** ***the value of the final Group Fund would not be reduced, so you would earn:***

**¼** final Group Fund, which would hold 80 tokens (100-20 tokens) that are worth 2 ECUs each = **¼** 160 ECUS = **40 ECUs**

***If the card drawn is equal to or smaller than 20 (which has a 20% probability) the value of the final Group Fund would be reduced by 90%, so you would earn:***

**¼** of 160 ECUs **- 90%** x 160 ECUs (144 ECUs)= ¼ 16 ECUs (160-144) = **4 ECUs**

**YOUR TOTAL EARNINGS WOULD BE DETERMINED AS FOLLOWS**

$$\left\{ \begin{matrix} \boldsymbol{if no reduction, 60 ECUs = 20}\boldsymbol{from your Individual Fund}\boldsymbol{+ 40}\boldsymbol{from the Group Fund} \\ \boldsymbol{if reduction, 24 ECUs = 20}\boldsymbol{from y}\boldsymbol{our Individual Fund}\boldsymbol{+ 4}\boldsymbol{from the Group Fund} \end{matrix} \right.$$

**Since none of the other participants moved any tokens to their Individual Funds, they would earn 0 ECUs from their Individual Funds. Plus, they would receive the same earnings as you from the Group Fund.**

**Please complete quiz 4 now**

**Quiz 4: Participant number ___________**

4.1. In Decision Situation 4 the starting value of your *Individual Fund* is ____ ECUs.

4.2. In Decision Situation 4, the starting value of the initial *Group Fund* is _____ ECUs.

4.3. In Decision Situation 4, each token you move from the initial *Group Fund* increases the value of your *Individual Fund* by ____ ECUs and reduces the value of the final *Group Fund* by _____ ECUs. In addition, each token a group member removes from the initial *Group Fund* increases by 1% the probability that the final *Group Fund* will reduce **______%** of its value.

**Decision Sheet PART A: Participant number ___________**

For each decision situation: **Write in the number of tokens you wish to move from the initial *Group Fund* to your *Individual Fund*.**

| **Decision Situations** | Number between 0 and 25 tokens you wish to move from the *Initial Group Fund* to your *Individual Fund.* | |
| --- | --- | --- |
|  |  |  |
| **Decision 1** | Tokens in the *Individual Fund* have a value of 1 ECU.  Tokens in the *Group Fund* have a value of 2 ECUs. | **____________ Token(s)** |
| **Decision 2** | Tokens in the *Individual Fund* have a value of 1 ECU.  Tokens in the *Group* Fund have a value of 2 ECUs  There is a 1% chance of **a 50%** reduction of the final *Group Fund* for every token moved from the *Group Fund*. | ____________ **Token(s)** |
| **Decision 3** | Tokens in the *Individual Fund* have a value of 1 ECU.  Tokens in the *Group Fund* have a value of 2 ECUs  There is a 1% chance of **a 10%** reduction of the final *Group Fund* for every token moved from the *Group Fund*. | ____________ **Token(s)** |
| **Decision 4** | Tokens in the *Individual Fund* have a value of 1 ECU.  Tokens in the *Group Fund* have a value of 2 ECUs  There is a 1% chance of **a 90%** reduction of the final *Group Fund* for every token moved from the *Group Fund*. | ____________ **Token(s)** |

**PART B: - Experiment Instructions**

In this section, you will be asked to **forecast** (for each decision situation) the **per person** **average number of tokens** (not including your decision) moved from your *Group Fund* to the *Individual Funds* of the other participants in your group (average: a number between 0 and 25). If you want, your forecast may include up to two decimals.

Your earnings for Part B will be determined in the following way: If your **forecast of the per person** **average number of tokens** moved from your *Group Fund* to the *Individual Funds* (for the chosen decision situation) is equal to or not more than 1 token away from the actual average, you will earn 5 euros. If your forecast is more than 1 token away from the average you will earn 2 euros divided by the (absolute) distance between your forecast and the actual average moved from the *Group Fund.*

After the four parts of the experiment and the survey are over, the monitor will randomly pick one of the 4 decision situations from part A for computing earnings. This same decision situation will be used for computing earnings for this part of the experiment, Part B. The draw will be made by picking a card out of a shuffled deck of cards numbered from 1 to 4. The drawing will be made in public, at the front of the room and it will be the same for all groups.

**Decision Sheet PART B: Participant number ___________**

| Per person average number of tokens moved by the other 3 members of your group from the *Group Fund* to the *Individual Fund* (**a number between 0 and 25**) | |
| --- | --- |
| **Decision 1:** *Group Fund* is reduced in value by 2 ECUS for each token moved to an *Individual Fund*. **There is no additional probability of reduction** of the final *Group Fund***.** | **_______________________** tokens |
| **Decision2**: *Group Fund* is reduced in value by 2 ECUS for each token moved to an *Individual Fund*. There is an **additional probability of reduction** of the final *Group Fund* **by 50%.** | **_______________________** tokens |
| **Decision 3:** *Group Fund* is reduced in value by 2 ECUS for each token moved to an *Individual Fund*. There is an **additional probability of reduction** of the final *Group Fund* **by 10%.** | **_______________________** tokens |
| **Decision 4:** *Group Fund* is reduced in value by 2 ECUS for each token moved to an *Individual Fund*. There is an **additional probability of reduction** of the final *Group Fund* **by 90%.** | **_______________________** tokens |

**PART C: - Experiment Instructions**

This part of the experiment consists of **10 decisions**. Each of your decisions is a **choice between the alternatives LEFT and RIGHT**. Decisions in Part C **have consequences only for your own payment. You are not in a group as you were in Part A.**

In each of the 10 decisions the alternative LEFT gives a sure payment. By contrast, your payment for the alternative RIGHT depends on chance.

| *Example:*  *You might be asked whether you prefer to choose alternative LEFT, in which you get* ***3.5 Euros for sure****, or alternative RIGHT, in which you have a* ***50% chance of getting 5 Euros*** *and a* ***50% chance of getting 0 Euros****. You have then to decide for one of the two alternatives.* *This decision problem would look like this on the decision sheet::*   \| **LEFT** \| **Your Choice (please mark)** \| **RIGHT** \| \| \| --- \| --- \| --- \| --- \| \| ***3.50 Euros*** for sure \| LEFT RIGHT \| 50% Chance of ***5 Euros***  and  50% Chance of ***0* Euros** \| |
| --- | --- | --- | --- | --- | --- | --- | --- |

Your total payoff from this part of the experiment is determined as follows: At the end of the experiment, **one of the 10 decision tasks** will be randomly chosen and the alternative chosen in this decision task will be **actually carried out and paid out**. The draw will be made by picking a card out of a shuffled deck of cards numbered from 1 to 10. The drawing will be made in public, at the front of the room and it will be the same for everyone in this session.

If, for example, the randomly chosen decision task was the one shown above, and if in this task you had chosen alternative RIGHT, then you would receive *5 Euros* with a probability of 50% and *0* Euros with a probability of 50%. I will once more make a random draw from a deck of cards numbered 1-10 to determine if you will be paid 0 or 5 euros. If an even number is chosen 0 will be paid, if an odd number is chosen, 5 will be paid.

**Decision Sheet PART C: Participant number ___________**

**Choose left or right for each decision.**

| **LEFT** | **Your Choice (please mark)** | **RIGHT** |
| --- | --- | --- |
| **you get** |  | **you get** |
| ***0.50 Euros*** for sure | LEFT RIGHT | 50% Chance of ***5 Euros***  and  50% Chance of ***0* Euros** |
| ***1.00 Euros*** for sure | LEFT RIGHT | 50% Chance of ***5 Euros***  and  50% Chance of ***0* Euros** |
| ***1.50 Euros*** for sure | LEFT RIGHT | 50% Chance of ***5 Euros***  and  50% Chance of ***0* Euros** |
| ***2.00 Euros*** for sure | LEFT RIGHT | 50% Chance of ***5 Euros***  and  50% Chance of ***0* Euros** |
| ***2.50 Euros*** for sure | LEFT RIGHT | 50% Chance of ***5 Euros***  and  50% Chance of ***0* Euros** |
| ***3.00 Euros*** for sure | LEFT RIGHT | 50% Chance of ***5 Euros***  and  50% Chance of ***0* Euros** |
| ***3.50 Euros*** for sure | LEFT RIGHT | 50% Chance of ***5 Euros***  and  50% Chance of ***0* Euros** |
| ***4.00 Euros*** for sure | LEFT RIGHT | 50% Chance of ***5 Euros***  and  50% Chance of ***0* Euros** |
| ***4.50 Euros*** for sure | LEFT RIGHT | 50% Chance of ***5 Euros***  and  50% Chance of ***0* Euros** |
| ***5.00 Euros*** for sure | LEFT RIGHT | 50% Chance of ***5 Euros***  and  50% Chance of ***0* Euros** |

**PART D - Experiment Instructions**

In this part of the experiment each of you will be paired with a set of charities. You will be making decisions for a total of 3 Euros; you must decide how many Euros (if any) you allocate to one or several of the charities below and how many Euros (if any) you take for yourself.

For every euro you allocate to a charity, we will transfer in addition 25 cents to the charity.

After all experimental sessions are over, on Friday November 30, the principal investigator of this project, Dr. Esther Blanco, will pool the money all participants have allocated to each one of the charities and will make an online payment for the sum of money plus 25 cents for every euro.

You will have available a list of all individual contributions to each charity (without participant name or number) as well as the total sum of money paid to each one of the charities on the personal webpage of Dr. Blanco.

**Decision Sheet PART D: Participant number ___________**

**Amount you wish to allocate to:**

WWF ____________

Doctors Without Borders ____________

Amnesty International ____________

SOS Kinderdorf ____________

Caritas ____________

Licht ins Dunkle ____________

Oxfam ____________

Red Cross ____________

YOU ____________

The sum must be equal to 3 euros.

**Official Mission Statements of the Organisations**

**WWF**'s mission is to conserve nature and reduce the most pressing threats to the diversity of life on Earth (<http://worldwildlife.org/about>).

**Doctors Without Borders** provides assistance to populations in distress, to victims of natural or man-made disasters and to victims of armed conflict (<http://www.doctorswithoutborders.org/aboutus/charter>).

**Amnesty International** is committed to the enforcement of the Universal Declaration of Human Rights and other rights which are part of international human rights agreements ([www.amnesty.at/ueber_amnesty](http://www.amnesty.at/ueber_amnesty)).

In the center of **SOS Children's Villages** is the effort to provide children who have lost their parents or can no longer live with them, with a lasting and permanent home and a stable environment ([www.sos-kinderdorf.at/sos-kinderdorf-erleben/unser-auftrag](http://www.sos-kinderdorf.at/sos-kinderdorf-erleben/unser-auftrag)).

Independent from their social, national or religious affiliation, **Caritas** supports and accompanies people in difficult life situations, who suffer from illness or disability as a consequence of accidents or disasters ([www.caritas.at/ueber-uns/leitbild](http://www.caritas.at/ueber-uns/leitbild)).

The philosophy of the organization "**Licht ins Dunkel** " is the material and emotional support of disabled children and their families, physically and mentally disabled people in Austria , as well as the promotion of the objectives of its members as set in the organization charta. ([http://lichtinsdunkel.orf.at](http://lichtinsdunkel.orf.at/)).

**Oxfam** is an independent relief and development organization. We are convinced that poverty and injustice are preventable and can be overcome ([www.oxfam.de/ueber-uns](http://www.oxfam.de/ueber-uns)).

The **Red Cross** mission is to improve the lives of people in need and vulnerable groups through the power of humanity ([www.roteskreuz.at/organisieren/organisation/wer-wir-sind](http://www.roteskreuz.at/organisieren/organisation/wer-wir-sind)).

**SURVEY Participant number ___________**

We appreciate your participation in this decision making experiment. To complete your participation please complete the following questionnaire. The questionnaire is anonymous and confidential.

1. In what year were you born?

2. Gender

Male  Female

3. How many siblings do you have?_____________________________

4. In which program are you enrolled in the University of Innsbruck? ____________________________________

5. Are you registered member of any charity or NGO?

Yes Which one(s)? ______________________________________________________

______________________________________________________

______________________________________________________

(if more, please continue in the bottom)

No

6. Did you participate in voluntary work in the last year?

Yes, approximately how many days in that year? __________________

For which organization?_______________________________________________________

No

7. Have you made a donation to any charity or NGO during the last year?

Yes Which one(s)? ____________________________________Amount:___________

___________________________________ Amount:___________

___________________________________ Amount:___________

(if more, please continue in the bottom)

No

8. How much do you trust in people from each of these groups?

|  | Fully trust | Mostly trust | Barely trust | Do not trust at all |
| --- | --- | --- | --- | --- |
| Your family | 1 | 2 | 3 | 4 |
| Your neighbours | 1 | 2 | 3 | 4 |
| Other students in UIBK | 1 | 2 | 3 | 4 |
| Someone I meet for the first time | 1 | 2 | 3 | 4 |

9. Is this the first decision experiment in which you have participated?

Yes

No

10. From 1 to 5 how clear were the instructions for Part A of the experiment?

Very Very

Clear Unclear

1  2  3  4  5

11. From 1 to 5 how clear were the instructions for Part B of the experiment?

Very Very

Clear Unclear

1  2  3  4  5

12. From 1 to 5 how clear were the instructions for Part C ofthe experiment?

Very Very

Clear Unclear

1  2  3  4  5

13. From 1 to 5 how clear were the instructions for Part D ofthe experiment?

Very Very

Clear Unclear

1  2  3  4  5

14. Did you have any questions you wanted to ask us? If yes, please briefly write them in the space provided

________________________________________________________________________________________________________________________________________________________________________________________________________________________________________________________________________________________________________________________________________________________________________________________________________________________________________________________________________________________________________________________________________________________________________________________________________________________________________________________________________
